# Identification of the protease cleavage sites in a reconstituted Gag polyprotein of an HERV-K(HML-2) element

**DOI:** 10.1186/1742-4690-8-30

**Published:** 2011-05-09

**Authors:** Maja George, Torsten Schwecke, Nadine Beimforde, Oliver Hohn, Claudia Chudak, Anja Zimmermann, Reinhard Kurth, Dieter Naumann, Norbert Bannert

**Affiliations:** 1 Center for HIV and Retrovirology, Robert Koch Institute, Nordufer 20, 13353 Berlin, Germany; 2 Project Group Biomedical Spectroscopy, Robert Koch Institute, Nordufer 20, 13353 Berlin, Germany; 3 Robert Koch Institute Fellow, Nordufer 20, 13353 Berlin, Germany; 4 Center for Biological Safety 4, Robert Koch Institute, Nordufer 20, 13353 Berlin, Germany

**Keywords:** HERV-K(HML-2), Gag processing, maturation, retrovirus, retroviral protease, endogenous retrovirus

## Abstract

**Background:**

The human genome harbors several largely preserved HERV-K(HML-2) elements. Although this retroviral family comes closest of all known HERVs to producing replication competent virions, mutations acquired during their chromosomal residence have rendered them incapable of expressing infectious particles. This also holds true for the HERV-K113 element that has conserved open reading frames (ORFs) for all its proteins in addition to a functional LTR promoter. Uncertainty concerning the localization and impact of post-insertional mutations has greatly hampered the functional characterization of these ancient retroviruses and their proteins. However, analogous to other betaretroviruses, it is known that HERV-K(HML-2) virions undergo a maturation process during or shortly after release from the host cell. During this process, the subdomains of the Gag polyproteins are released by proteolytic cleavage, although the nature of the mature HERV-K(HML-2) Gag proteins and the exact position of the cleavage sites have until now remained unknown.

**Results:**

By aligning the amino acid sequences encoded by the *gag-pro-pol *ORFs of HERV-K113 with the corresponding segments from 10 other well-preserved human specific elements we identified non-synonymous post-insertional mutations that have occurred in this region of the provirus. Reversion of these mutations and a partial codon optimization facilitated the large-scale production of maturation-competent HERV-K113 virus-like particles (VLPs). The Gag subdomains of purified mature VLPs were separated by reversed-phase high-pressure liquid chromatography and initially characterized using specific antibodies. Cleavage sites were identified by mass spectrometry and N-terminal sequencing and confirmed by mutagenesis. Our results indicate that the *gag *gene product Pr74^Gag ^of HERV-K(HML-2) is processed to yield p15-MA (matrix), SP1 (spacer peptide of 14 amino acids), p15, p27-CA (capsid), p10-NC (nucleocapsid) and two C-terminally encoded glutamine- and proline-rich peptides, QP1 and QP2, spanning 23 and 19 amino acids, respectively.

**Conclusions:**

Expression of reconstituted sequences of original HERV elements is an important tool for studying fundamental aspects of the biology of these ancient viruses. The analysis of HERV-K(HML-2) Gag processing and the nature of the mature Gag proteins presented here will facilitate further studies of the discrete functions of these proteins and of their potential impact on the human host.

## Background

During the early and more recent evolution of our primate and hominid ancestors, a number of retroviruses infected the germ line cells, thereby becoming vertically transmitted genetic elements [1]. Today these so-called Human Endogenous Retroviruses (HERVs) constitute approximately 8% of our genome [2]. One likely reason for this accumulation is the inability of the host cell to reverse the retroviral integration process. Although long neglected as junk DNA, evidence is now accumulating that several elements, at least, are involved in certain physiological and pathological processes [2-5]. HERVs are known to regulate the expression of several genes and two HERV envelope proteins (syncytins) are involved in placental development [6,7]. The discovery of endogenous retroviral particles in cancer cells, as well as their similarity to exogenous cancer-inducing retroviruses, prompted intense interest in these ancient viruses and their possible association with malignant transformation [8-10]. Although during the course of evolution many HERVs have accumulated a number of post-insertional mutations (simply by copy errors made by the host DNA polymerase) as well as extensive deletions, some have retained open reading frames (ORFs) for viral proteins such as the group specific antigen (Gag) [11,12]. However, none of these virtually complete proviruses has been shown to be fully functional and replication competent. The betaretrovirus HERV-K(HML-2) family of endogenous human retroviruses is the best preserved and most recently active, having first entered the germ lines of human predecessors as exogenous retroviruses about 35 million years ago [13]. The presence of several exclusively human proviral elements indicates ongoing activity less than 5 million years ago, after the split of the human and chimpanzee lineages [14-16].

Recently, two synthetic consensus sequences based on the alignment of a number of human-specific members of the HERV-K(HML-2) family were constructed [17,18] and shown to be able to produce infectious retrovirus-like particles. Using a similar approach we have reconstituted the original envelope protein of one of the youngest HERV-K(HML-2) elements, HERV-K113, and demonstrated its restored functionality [19]. There is no evidence that the HERV-K113 element suffered from the action of the APOBEC family of proteins [20]. In the present study we identified non-synonymous post-integrational mutations in the *gag-pro-pol *region of the HERV-K113 sequence present in a BAC library clone [14,15] and reconstituted the original ancient Gag precursor proteins. This reversion of the post-insertional mutations made it possible to investigate the cleavage of the HERV-K(HML-2) Gag precursor protein during viral maturation.

The internal structural proteins of all retroviruses, including ancient betaretroviruses, are synthesized as large Gag polyproteins [21]. In addition, the position of the reading frames in the proviral sequence of HERV-K(HML-2) indicates that ribosomal frameshifting is necessary for the synthesis of the Gag-Pro and Gag-Pro-Pol polyproteins as has been shown for the closely related mouse mammary tumour virus (MMTV) [22]. The three types of Gag polyproteins oligomerize and form roughly spherical immature virions which bud from the cell membrane, independent of envelope proteins [23]. During egress or shortly thereafter, the Gag, Gag-Pro and Gag-Pro-Pol polyproteins in the immature particle are cleaved by the viral protease (PR). Cleavage leads to the dramatic morphological changes known as maturation and renders the virus infectious. During this process the Gag protein itself is further cleaved by the protease to yield the major mature proteins matrix (MA), capsid (CA) and nucleocapsid (NC). The capsid protein is the main structural element of the mature virus particle, forming a core shell around the NC-RNA complex, while MA remains bound to the viral lipid bilayer. Depending on the genus of the virus, additional proteins and peptides are also released. In the case of MMTV, these are the polypeptides pp21, p8, p3 and n located between MA and CA [24]. These proteins appear to play a role in Gag folding, intracellular transport, assembly or maturation, although their precise functions are still poorly understood [25].

Several HERV-K(HML-2) proviruses encode functional PR proteins, an enzyme that has previously been expressed and partially characterized [26-29]. Although proteolytic Gag fragments have been described in teratocarcinoma cells expressing HERV-K and found to be released from *in vitro *translated Gag proteins following incubation with recombinant PR, the precise nature of these protein domains and their cleavage sites remains open [26,28,30].

In this report, we identify the processing sites in the Pr74^Gag ^of this primordial betaretrovirus. Similar to MMTV, the Mason-Pfizer monkey virus (MPMV) and other closely related viruses, HERV-K(HML-2) also encodes additional polypeptides between the MA and CA subdomains. We identified a 14 amino acid long spacer peptide, SP1, adjacent to the MA domain and a subsequent 15 kDa protein (p15). Moreover, two short glutamine- and proline-rich peptides are released from the C-terminus of the polyprotein. Our results using this archival virus further contribute to the understanding of retroviral Gag processing and maturation. The exact identification of the Gag subdomains in this paper is a prerequisite for their accurate molecular cloning or the generation of deletion mutants. It facilitates the characterization of post-translational modifications in the subunits and will help future studies into their role during assembly and other replication steps. In this regard, the role of the two C-terminal QP-rich peptides reported here will be of particular interest. The results also allow the unequivocal localisation of functional domains, e.g. L-domains, to individual Gag subunits.

## Results

### Reconstitution of the *gag-pro-pol *coding region of the original HERV-K113 provirus and expression of a partially codon optimized sequence

Expression levels of the Gag protein and virus-like particles of the native HERV-K113 sequence in transfected cells are very low, making detection difficult [30-32]. This is mainly the result of mutations in the proviral DNA acquired after insertion into the host's genome [19,31] and the use of rare codons by the virus. To overcome this obstacle, we employed the same approach previously described to reconstitute and express the original envelope protein of HERV-K113 [19] at high levels. To identify post-insertional mutations in the HERV-K113 *gag-pro-pol *region, we aligned the amino acid sequences encoded by the ORFs with those of 10 well-preserved human specific HERV-K(HML-2) viruses (Additional File 1A). If none or only one of the other elements had the same amino acid at a certain position, the underlying nucleotide difference was assumed to have been introduced into HERV-K113 after insertion. If two or more of the elements shared a difference with HERV-K113 (even if different from the consensus sequence), it was considered to be a shared polymorphism already present at the time of integration and was therefore left unchanged. In total, 5 putative protein-relevant post-insertional mutations were identified in the Gag protein, 3 in the ORF of the PR and 8 in the ORF of the polymerase (Additional File 1A).

**Figure 1:**
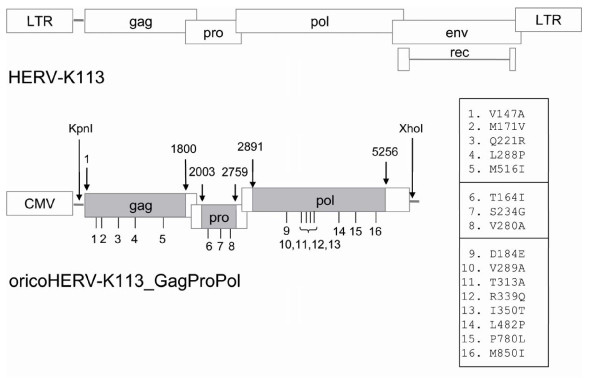
**Schematic representation of the HERV-K113 provirus and structure of the oricoHERV-K113_GagProPol construct**. To express high levels of the original HERV-K113 Gag, Gag-Pro and Gag-Pro-Pol proteins, a partially codon-optimized sequence (gray areas) encoding the reconstituted amino acid sequence of the virus was cloned downstream of the CMV promoter in the pcDNA3.1 vector. The 16 identified and reverted post-insertional amino acid changes are listed next to the oricoHERV-K113_GagProPol structure. Their positions in the open reading frames are indicated underneath. Numbers above refer to nucleotide positions of the codon-optimized regions starting with the first nucleotide of *gag*.

To enhance the expression of the Gag, Gag-Pro and Gag-Pro-Pol proteins, large sections of the viral DNA encoding the three reconstituted proteins were codon-optimized for mammalian cells. Regions corresponding to slippery sites and overlapping ORFs (Figure 1) were kept in their native form to allow frame shifts for the expression of the protease and polymerase. The synthetic sequence (oricoHERV-K113_GagProPol) was cloned in the pcDNA3.1 expression vector to allow CMV-promoter driven expression (Additional File 1B). The prefix orico is derived from the abbreviation 'ori' (reversion of post-insertional mutations into the *original *amino acid sequence) and 'co' for codon optimization.

### Production of maturation-competent VLPs by expression of reconstituted HERV-K113 Gag polyproteins

**Figure 2:**
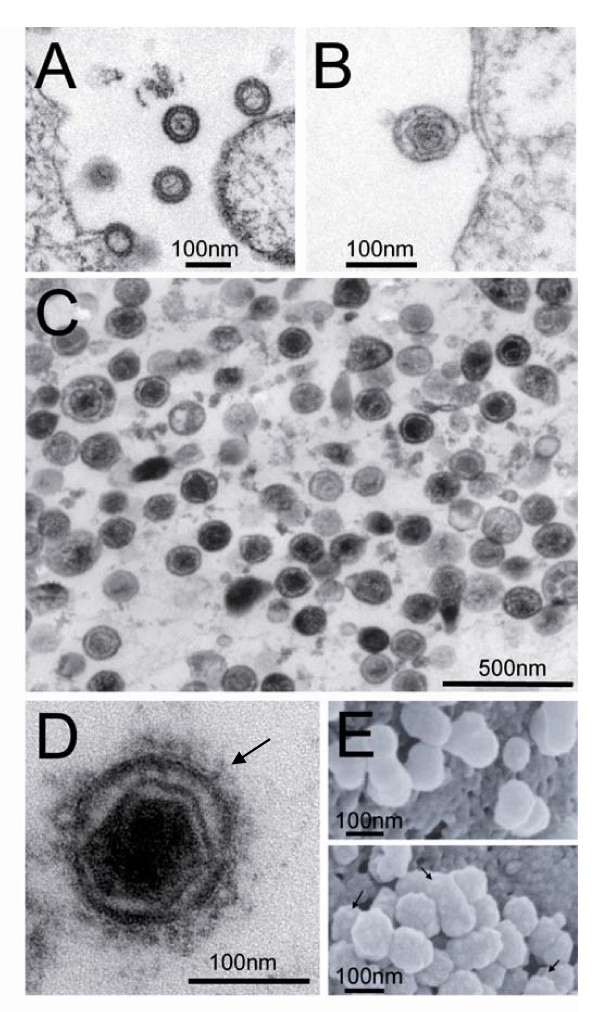
**Electron microscopic analysis of the oricoHERV-K113_GagProPol VLP morphology**. (A) Immature particles budding from the cell and being released. (B) Particles with condensed cores can be observed close to the cell membrane demonstrating an active protease and the ability of the VLPs to mature. (C) Thin section micrograph of a pellet made by ultracentrifugation of supernatants from VLP-producing cells. All VLPs show condensed cores. (D) Transmission electron microscopy of VLPs showing incorporation of a reconstituted HERV-K113 envelope protein [19] expressed *in trans*. The arrow indicates the Env proteins on the surface of the virion. (E) Scanning electron microscopy of VLPs at the surface of cells. The upper panel shows VLPs produced with pcDNAoricoHERV-K_GagProPol and the lower panel VLPs with reconstituted Env at the surface (arrows) which was expressed *in trans*.

The ability of oricoHERV-K113_GagProPol to generate VLPs was investigated by electron microscopy (EM). HEK 293T cells were transfected and incubated for two days before harvesting cells and supernatants. Viral particles were purified from supernatants by ultracentrifugation and cells and virus pellets were then prepared for thin section EM. Immature virions with an electron dense ring structure (Figure 2A) as well as mature particles with an electron dense core (Figure 2B) were observed at the cell surface, whereas virus pellets consisted exclusively of mature virions (Figure 2C). By co-expressing a reconstituted HERV-K113 envelope protein [19]*in trans *it was possible to show by transmission electron microscopy (Figure 2D) and scanning electron microscopy (Figure 2E) that the protein can be incorporated into the VLPs. Moreover, the supernatant of cells expressing the VLPs contain reverse transcriptase activity as measured using the Cavidi RT-Assay (data not shown).

### Identification and characterization of the major mature HERV-K(HML-2) Gag proteins

**Figure 3:**
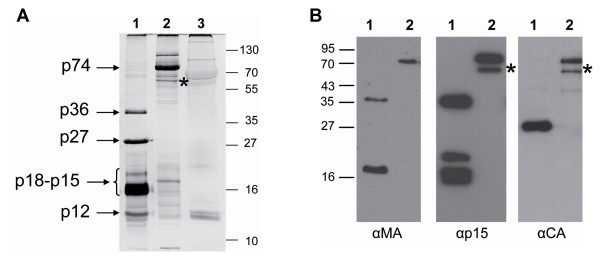
**Detection of major Pr74^Gag ^processing fragments by SDS-PAGE and Western blotting**. Viral particles produced in HEK 293T cells were purified by ultracentrifugation through a 20% sucrose cushion and the pellets loaded on 15% gels. (A) Silver-stained SDS-PAGE. Lane 1: VLPs produced with oricoHERV-K113_GagProPol. Lane 2: VLPs produced by a mutant with an inactive protease (oricoHERV-K113_GagPro^-^Pol). Lane 3: Empty vector control. (B) Western blot analysis of the VLPs. Lane 1: oricoHERV-K113_GagProPol. Lane 2: oricoHERV-K113_GagPro^-^Pol (PR-mutant). The blots were probed using antisera generated against recombinant proteins of predicted MA (left panel), p15 (central panel) and CA (right panel) polypeptides of HERV-K113. The band marked with a star is an unspecific N-terminal degradation product of the Gag precursor that accumulates in the protease-deficient mutant. M, molecular mass marker.

We next analyzed proteins in the virus pellets by silver nitrate stained sodium dodecyl sulphate polyacrylamide gel electrophoresis (SDS-PAGE). In addition to the oricoHERV-K113_GagProPol, cells were also transfected with a maturation defective mutant (oricoHERV-K113_GagPro^-^Pol) carrying PR inactivating D204A, T205A and G206A mutations in the active site of the enzyme. A protein migrating with an apparent molecular mass of 78 kDa, corresponding well to the expected size of the HERV-K(HML-2) Gag precursor protein (74 kDa), was present in pellets of the PR mutant. Such a band was absent or barely visible in pellets of reconstituted VLPs carrying an active PR (Figure 3A). Here, bands of 36 kDa, 27 kDa, 15-18 kDa and 12 kDa, presumably processed Gag polypeptides, were exclusively present in these pellets (Figure 3A, lane 1). Expression of the reconstituted proteins encoded in the *gag-pro-pol *region of HERV-K113 therefore leads to the production and release of maturation competent VLPs.

A comparison of the HERV-K(HML-2) Gag sequence with those of other betaretroviruses suggests that in addition to the canonical matrix (MA), capsid (CA) and nucleocapsid (NC) proteins, at least one further polypeptide of approximately 15 kDa (designated here as p15) might be encoded between the MA and CA domains. Such protein(s) are known to exist in the closely related MMTV and MPMV viral particles [24,33].

In an attempt to assign the mature Gag proteins observed to the expected MA, p15, CA and NC processing products we generated a series of specific antisera by immunizing rats with *E. coli*-expressed fragments of the Pr74^Gag ^protein. An antiserum raised against amino acids 1-100 (αMA), expected to include the MA protein, reacted with a 36 kDa and a 16 kDa protein (Figure 3B). A second antiserum (αp15), specific for amino acids 140-282, also recognized the 36 kDa protein and a triplet of bands in the 15-18 kDa region (Figure 3B). The ratio of intensities between the triplet bands and the 36 kDa band varied to some extent, depending on the preparation. Since the 36 kDa protein was detected by the αMA and the αp15 antisera, we assume that this protein represents a processing intermediate comprising the approximately 16 kDa MA and the p15 protein. Finally, a single band of 27 kDa was detected using an antiserum (αCA) specific for amino acids 283-526, presumably corresponding to the CA subdomain. All three antisera reacted with the unprocessed Gag precursor expressed by the inactive PR mutant (Figure 3B).

**Figure 4:**
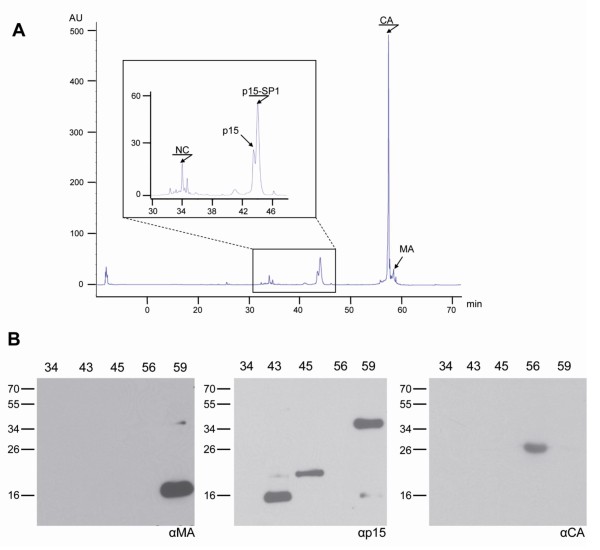
**Separation of Pr74^Gag^ cleavage  products by RP-HPLC.** (A) Gag subdomains of purified HERVK113_  GagProPol VLPs were  chromatographically separated by RPHPLC  on an RP-C8 column. Proteins  were eluted by an increasing acetonitrile  gradient. Fractions were taken every  minute and the eluted material was  detected by UV absorption at 280 nm  (AU, adsorption units). (B) The proteins  in the fractions with the major peaks  (fraction 34, 43, 45, 56 and 59) were  analyzed by Western blot using the  antisera against the presumed MA (left  panel), p15 (central panel) and CA (right  panel) domains.

To further delineate the nature of the processed HERV-K(HML-2) Gag domains, we separated the proteins from mature VLPs by HPLC on a reverse phase column. Fractions of 500 μl were collected and the eluted proteins detected by UV absorption at 280 nm (Figure 4A). Fractions containing the major protein peaks were then analysed by Western blot using the antisera described above (Figure 4B). The assumed 16 kDa MA protein, recognized by the rat αMA serum, was present together with traces of the 36 kDa protein in fraction 59 (Figure 4B, left panel). The proteins in this fraction were also recognized by the HERMA4 monoclonal antibody [30] indicating that it binds to an epitope within the MA domain (data not shown). The αp15 antiserum also detected the 36 kDa protein, providing further evidence that this is a processing intermediate containing MA-p15. The smallest fragment of the 15-18 kDa triplet recognized by the αp15 antiserum was eluted in fraction 43 and the largest mainly in fraction 45 (Figure 4B, middle panel). These two protein bands were usually the strongest of the triplet. The commercially available monoclonal antibody HERM-1841-5 (Austral Biologicals) reacted with the same proteins, indicating that its epitope is located in the p15 protein (data not shown). The presumed 27 kDa CA protein was detected in fraction 56 (Figure 4B, right panel). None of the antisera reacted with proteins in fraction 34.

### Determination of protease cleavage sites by N-terminal sequencing of isolated HERV-K(HML-2) Gag proteins

**Figure 5:**
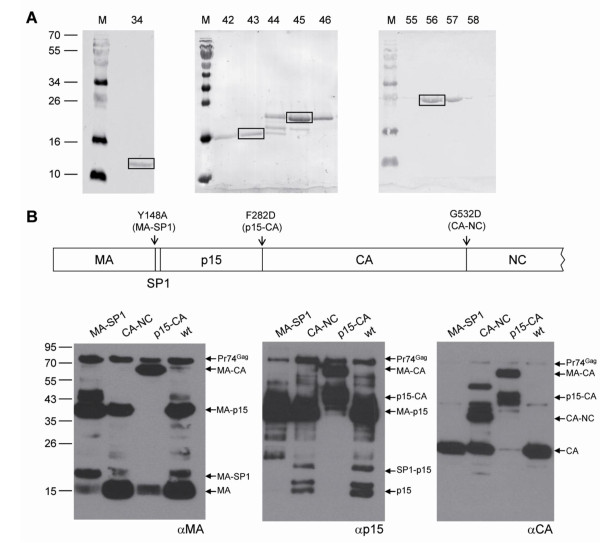
**Cleavage site determination by Nterminal  sequencing**. (A) Proteins from RP-HPLC fractions known to  contain mature Pr74Gag subdomains  were blotted on a PVDF membrane and made  visible by staining with Ponceau S. Protein bands  corresponding to the specific sizes of processed  Gag domains were cut out (bands framed with  black boxes) and sent for Edman degradation to  determine the N-terminal amino acid sequence. (B)  Western blot analysis of oricoHERVK113_  GagProPol mutants carrying amino acid changes at  the P1 position of the MASP1 site (Y134A, lane  1), the CA-NC site (G532D, lane 2) and the p15-  CA site (F282D, lane 3). VLPs with wild type (wt)  cleavage sites were run in lane 4.

The fractions containing diverse p15 fragments (fractions 43-46), the CA protein (fraction 55-58) and the putative NC protein (fraction 34) were subjected to SDS-PAGE, transferred to PVDF membranes and stained with Ponceau S (Figure 5A). The major bands on the membrane corresponded with the molecular mass of the proteins previously identified by specific antisera. Fractions 43 to 46 gave two major bands migrating with apparent molecular masses of 15 kDa and approximately 18 kDa as well as an additional weaker band between these two. The two major proteins of this subdomain, the CA protein of fraction 56 and the assumed NC protein of 12 kDa in fraction 34, were cut out and N-terminally sequenced (Figure 5A). The N-terminal sequences obtained by Edman degradation confirmed the identity of the processed Gag subdomains and identified the cleavage sites (Table 1). This also allowed the calculation of the theoretical molecular masses of the released proteins. Sequencing also revealed that the N-termini of the two p15 variants differ by the 14 amino acid peptide "VAEPVMAQSTQNVD" which we have designated 'spacer peptide 1' (SP1). To address the possibility that the p15 variants also vary at their C-termini, each was digested with trypsin and the fragments analyzed by MALDI-TOF. In both samples, a peptide of 1210.3 Da was detected, corresponding to the C-terminal trypsin-digested fragment "KEGDTEAWQF" (theoretical average molecular weight 1210.3 Da) preceding the N-terminal CA sequence (data not shown). The sequence of this C-terminal p15 peptide was further verified by MALDI-TOF MS/MS (data not shown). These experiments confirm that the two p15 variants share the same C-terminal sequence. The larger p15 protein with a calculated molecular mass of 16.5 kDa (18 kDa on SDS-PAGE) is therefore a cleavage intermediate from which a 14 amino acid peptide (SP1) of 1.5 kDa is released to generate the mature 15 kDa p15 protein (15-16 kDa on SDS-PAGE).

**Table 1:** Cleavage sites identified by N-terminal sequencing of purified Pr74^Gag ^subdomains

**Subdomain**	** P4 **	**P3**	** P2**	** P1**	** - **	**P1'**	** P2'**	** P3'**	**P4'**	** P5'**
MA - SP1	His-	Cys-	Glu-	Try-	:	-Val	-Ala	-Glu	-Pro	-Val
SP1 - p15	Gln-	Asn-	Val-	Asp-	:	-Try	-Asn	-Gln	-Lys	-Gln
p15 - CA	Ala-	Trp-	Gln-	Phe-	:	-Pro	-Val	-Thr	-Lys	-Glu
CA - NC	Ala-	Iso-	Thr-	Gly-	:	-Val	-Val	-Lys	-Gly	-Gly

Amino acid sequences determined by N-terminal sequencing are underlined.

### Validation of the cleavage sites identified by N-terminal sequencing

In retroviral cleavage sites, the P1 position (amino acid preceding the scissile bond) is generally hydrophobic and unbranched at the β-carbon [34]. This principle is fulfilled in all cleavage sites identified here with the exception of the SP1-p15 site. The Asp in P1 renders this position rather unlikely to be a retroviral PR cleavage site [34]. To test whether an Asp in P1 inhibits hydrolysis by the HERV-K(HML-2) PR, we substituted the hydrophobic residues in the P1 positions of the p15-CA and CA-NC cleavage sites for Asp. We also substituted Tyr for Ala at the P1 position of the cleavage site used to release the mature MA protein. This resulted in a dramatic reduction in the extent of cleavage at this site with only a residual amount of mature 16 kDa MA being observed (Figure 5B). The amount of an 18 kDa protein, consistent with the MA-SP1 intermediate that is usually barely visible in wild type VLPs, increased accordingly. Introduction of an Asp at the P1 position of the canonical type I cleavage site between p15 and CA not only prevented cleavage at this site but also severely impaired processing at other sites. This resulted in the presence of far more MA-SP1-p15-CA precursor than mature MA protein (Figure 5B). Interestingly, substitution of Gly for Asp at the P1 position of the CA-NC scissile bond, a canonical type II cleavage site [34], only partially inhibited processing, with a significant release of the mature 27 kDa CA protein still occurring. This indicates that an Asp at the P1 position of at least some type II cleavage sites is possible, the hydrolysis however seems to be inefficient and slow.

### Further processing at the C-terminus of the Pr74^Gag ^precursor results in the release of two glutamine- and proline-rich polypeptides

**Figure 6:**
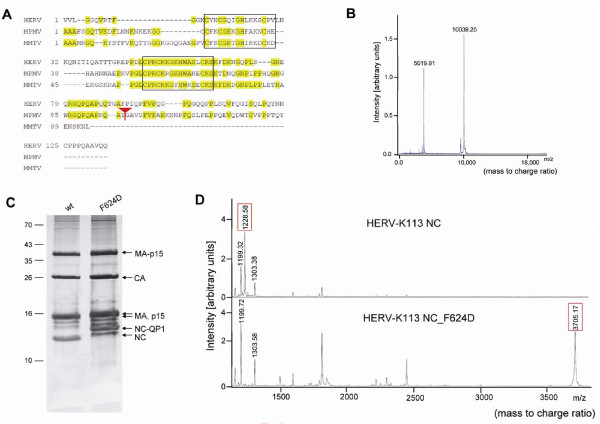
**Characterization of the processing at the Pr74^Gag ^C-terminus**. (A) Alignment of the amino acid sequences of oricoHERV-K113, MPMV (AAC82573) and MMTV (AAC82557.1) starting from the N-terminus of the NC domains. The red arrow indicates the NC-p4 cleavage site in MPMV [24]. Identical amino acids in different sequences are indicated in yellow. Black boxes span the RNA-binding zinc finger region. The alignment was generated using BLOSUM 62 (Clone Manager) and was subsequently adjusted by hand. (B) MALDI-TOF analysis of the NC domain of oricoHERV-K113. The first major peak represents doubly charged NC (z = 2) and the second major peak NC with a single charge. (C) Confirmation of the C-terminal NC cleavage site by mutagenesis. HERV-K113 VLPs and F624D mutants were loaded on an 18% SDS-PAGE and protein bands visualized by silver nitrate staining. (D) MALDI-TOF analysis of wt NC and the F624D mutant. The NC subdomains were purified by RP-HPLC and trypsin digested before MALDI-TOF analysis. Peaks of the wt and of the F624D mutant are shown in the upper and lower spectra respectively. The major peak of 1228.58 Da (framed) is unique for wt and the 3705.17 Da peak (framed) is unique for the F624D mutant. These peaks match with the sequences "GQPQAPQQTGAF" in the wt NC digest and "GQPQAPQQTGADPIQPFVPQGFQGQQPPLSQVFQG" in the F624D mutant. The peaks of approximately 1199 and 1303 Da visible in both spectra match with the expected trypsin fragments "QNITIQATTTGR" and "NGQPLSGNEQR" from internal NC regions. Additional peaks could be assigned to trypsin generated NC peptides (not shown).

The apparent molecular mass of the presumed mature NC protein on SDS-PAGE (12 kDa, see Figure 3A) was lower than the calculated value (14.6 kDa) and a comparison of the Gag C-termini of HERV-K113, MMTV and MPMV indicated that HERV-K(HML-2) might also release a C-terminal Gag polypeptide (Figure 6A) similar to the MPMV p4 subdomain [24]. Such a polypeptide would be highly glutamine- and proline (QP)- rich. This was supported by MALDI-TOF measurements of the NC subdomain, which yielded a molecular mass of only 10 kDa (Figure 6B).

To identify further processing sites at the C-terminus of the Gag-precursor, a tryptic digest of the NC subdomain (fraction 34 of the RP HPLC run) was subjected to MALDI-TOF analysis. This identified a "GQPQAPQQTGAF" peptide of 1228.58 Da that although being cleaved by trypsin at the N-terminus could not have been formed by trypsin cleavage at the C-terminus and therefore represented the C-terminus of the mature NC subdomain. Cleavage by the viral PR at this site not only generates a NC of 10 kDa but also corresponds well to the region in which NC-p4 cleavage in the MPMV Gag protein occurs (Figure 6A). However, it was not possible using SDS-PAGE or reverse phase-HPLC of VLPs to identify the expected C-terminal QP-peptide of 4.6 kDa.

**Figure 7:**
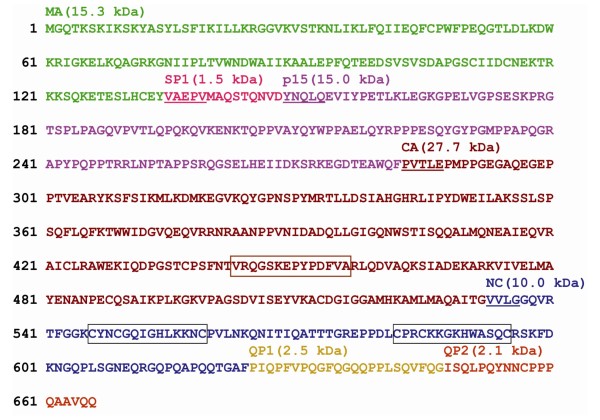
**Localisation of the protease cleavage sites in the Gag precursor protein of HERV-K113**. Amino acid sequence of Pr74^Gag ^depicting all processing sites and the molecular masses of the subdomains. The frame in the CA subdomain indicates the major homology region. The frames in NC indicate the CCHC-boxes.

Subsequently an Asp was introduced at the P1 position of the C-terminal NC cleavage site (F624D mutation) to block or at least impair the release of the expected C-terminal peptide. Because an NC-specific antiserum was not available, the effect of this mutation was initially investigated using SDS-PAGE. Unexpectedly, the mutation shifted a large fraction of the NC protein only by about 2.5 kDa (Figure 6C) and not the expected 5 kDa, which would have been consistent with the remaining C-terminal sequence attached to the NC. The mutant protein was therefore purified by RP-HPLC and analyzed by MALDI-TOF, which indicated a molecular mass of 12.5 kDa (data not shown). A tryptic digest generated the anticipated NC subunit fragments but, as expected, did not contain the "GQPQAPQQTGAD" peptide. Instead, the same peptide with a 23 amino acid extension was detected (Figure 6D). The F624D mutation therefore confirmed the C-terminal NC processing site identified earlier and revealed a further cleavage site in the C-terminal QP-rich sequence. Therefore, a 23 amino acid-long QP-rich peptide 1 (QP1) and a 19 amino acid-long QP-rich peptide 2 (QP2) are released from the C-terminus of the Pr74^Gag ^protein. All processing sites, molecular masses and subdomain sequences of the reconstituted HERV-K113 Gag precursor protein are depicted in Figure 7.

## Discussion

The ability of some human endogenous retroviruses to produce viral particles has been known for many years [11,35], and such virions have been shown to be expressed in a variety of tumour cells, including teratocarcinomas and melanomas. These proviruses generally belong to the HERV-K(HML-2) family, which includes the most recently integrated human endogenous elements. All known HERV-K(HML-2) proviruses have acquired multiple inactivating mutations or deletions after integration into the host chromosomes, although this does not rule out the possibility of infectious viruses emerging by recombination or of functional proviruses existing at a low prevalence within some human populations [17,36].

Despite accumulating evidence and growing interest in the oncogenic and other pathogenic aspects of HERV-K(HML-2)-encoded proteins, numerous fundamental properties of these ancient retroviruses remain virtually unknown. Studies of the virus and its proteins have been complicated or even prevented by its many incapacitating deletions and mutations. Recently however, the generation of two infectious HERV-K(HML-2) genomes based on consensus sequences and the reconstruction of the original HERV-K113 envelope gene have made it possible to express functional viral proteins and particles and hence study their properties [17-19]. Here, we used a procedure already successfully employed to reconstitute the envelope protein of HERV-K113 [19] to 'repair' the *gag-pro-pol *region of the virus. This method involves the identification and reversion of non-synonymous post-insertional mutations and allows discrimination between these positions and variations shared by a minority of the fossil elements. It, therefore, yields a protein sequence likely to be identical or very close to that of the virus existing at the time of integration approximately one million years ago [37]. To enhance expression of the Gag precursor protein, we generated a synthetic and partially codon-optimized sequence and cloned it under the control of the CMV promoter.

Thin section electron microscopy revealed that cells transfected transiently released a large number of retroviral particles. The presence of immature VLPs (with an opaque ring surrounding a relatively electron-lucent interior) and mature VLPs (with collapsed electron dense cores) suggested the activity of a functional protease and the completion of a regular maturation process. In contrast to recently budded particles located close to cells, pelleted supernatants only contained virions with spherical cores. This indicates that the vast majority of particles undergo maturation after release from the cell, but that it is somewhat delayed compared with other retroviruses e.g. HIV. Whereas several processed viral proteins were detected in the pellets of cells expressing the reconstituted and partially codon-optimized *gag-pro-pol *construct, only the 74 kDa Gag-precursor [32] was present in the supernatants of cells expressing a protease defective mutant. Immunoblotting with a combination of polyclonal sera raised against predicted domains of the HERV-K113 Gag protein and previously described monoclonal antibodies confirmed that most of the major bands from viral pellets are Gag processing fragments and provided some preliminary information concerning their identity. The cleavage fragments were further purified and separated by reverse phase HPLC and, with the help of the specific sera and antibodies, the fractions containing MA, CA and variant forms of a p15 protein, presumed to reside between MA and the CA domain, were identified. The identities of the p15 variants and the CA and NC proteins were subsequently confirmed by N-terminal Edman sequencing and mass spectrometry. N-terminal sequencing identified the exact locations of the cleavage sites releasing these domains and the sites were subsequently confirmed by mutagenesis. Moreover, mass spectrometry of the assumed NC subdomain provided strong evidence for a further cleavage that eliminates a C-terminal glutamine- and proline-rich sequence of 42 amino acids (QP-rich peptide) from the NC. A cleavage block introduced at this position corroborated this and revealed a further processing site that divides the 42 amino acid-long sequence into the QP1 and QP2 peptides of 23 and 19 amino acids respectively. As no such intermediate was detectable, cleavage at the NC-QP1 site seems to be relatively rapid. All cleavage sites identified were found to be highly conserved between HERV-K(HML-2) elements. Regarding the post-insertional mutations of the HERV-K113 haplotype that was used for the reconstitution, only the A147V mutation is close enough to a cleavage site that it may considerably effect processing. However, Ala and Val are both hydrophobic and not branched; therefore, a significant impact on the cleavage efficiency is rather unlikely [34]. Previously it has been shown that the I516M mutation in this element blocks particle formation and therefore replication [31].

The mechanism of site selection by retroviral PR remains poorly understood. It is known that structural requirements and approximately seven residues (positions P4 through P3') of the substrate define a scissile bond [34,38]. About 80% of all known PR cleavage sites can be classified into one of two groups. Type 1 sites have an aromatic residue in P1 and Pro at the P1' position while type 2 sites have a hydrophobic residue (excluding Ile and Val) at P1 and prefer Val, Leu or Ala at P1' [34,38,39]. Moreover, a type 1 site is always located at the N-terminus of CA and a type 2 site is usually present at the C-terminus of this domain. Our results demonstrate that this is also true for the CA protein of HERV-K(HML-2). The CA domain of HERV-K113, which forms the core of the mature virus, has a calculated molecular mass of 27.7 kDa and a HERV-K(HML-2) Gag cleavage product of comparable size has been described previously [18,28].

The mature 10 kDa NC, adjacent to CA, contains motifs for two zinc finger RNA binding domains (Cys-X_2_-Cys-X_4_-His-X_4_-Cys). It is interesting that the P1 and P1' positions (Phe-624 and Pro-625) of the NC-QP1 scissile bond define it as type 1 and that both positions match exactly with the site producing the N-terminus of CA. Furthermore, type 1 cleavage sites are also present at the NC-p6 junction in HIV-1/HIV-2 as well as at the NC-p9 site of Equine infectious anaemia virus (EIAV). In contrast, the QP1-QP2 cleavage site is of type 2.

In addition to the QP-rich peptides, two major forms (15 and 16.5 kDa) of an additional non-canonical protein that is encoded between the MA and CA subdomains were identified. These differ at the N-terminus by a peptide of 14 amino acids. We infer that this is a spacer peptide (SP1) present at analogous positions in other retroviruses whose function it is to regulate the maturation process [24,33,40]. The p16.5 protein is an SP1-p15 processing intermediate and the p15 represents the mature subdomain. This conclusion is in agreement with results obtained by inhibiting MA-SP1 cleavage in a P1 site mutant, which led to the accumulation of a slightly larger MA protein consistent with the size of the MA-SP1 fragment. The mutated cleavage site between the MA protein and the presumed spacer peptide SP1 fulfils the requirements for a type 2 site. The mature MA protein of our prototypical HERV-K(HML-2) has a calculated molecular mass of 15.3 kDa. This is somewhat larger than the 11 kDa MA protein of the closely related betaretroviruses MMTV and MPMV (11.9 kDa) [41]. In contrast to the other Gag cleavage sites identified here, the SP1-p15 site is neither of type 1 nor of type 2. The hydrophilic Asp residue in the P1 position of the scissile bond and the Tyr in P1' do not conform to the sequence of a conventional PR cleavage site. This raises the question of whether the retroviral PR or a cellular peptidase is responsible for the activity at this site. Such a peptidase could be a contaminant or be part of the virion. Particle-bound cellular peptidases engaged in Gag processing have been reported previously for at least murine leukaemia virus (MLV) and Rous sarcoma virus (RSV) [42,43]. Although we cannot exclude that a particle-associated or contaminating cellular peptidase is responsible for the cleavage or trimming at the atypical SP1-p15 site, we could demonstrate by changing the Gly to Asp in the P1 position of the CA-NC site (a type 2 site) that the HERV-K113 PR can in principle tolerate a hydrophilic Asp residue at P1. Although, compared to the wild type, cleavage of this mutant was only partially reduced, an Asp at the P1 position of the p15-CA site (a type 1 site) almost completely abrogated hydrolysis. Therefore, an Asp at P1 does not preclude PR processing *per se*, but there are other factors that determine cleavage effectiveness as well. In a very recently publication the cleavage at this site has also been documented investigating the Gag processing of the HERV-K_CON _consensus virus [44]. Further studies are needed to precisely characterize the processing at this site. The proteins between MA and CA in beta-, gamma-, delta and alpharetroviruses are often phosphorylated. The protein bands between the p15 and p16.5 variants observed on SDS-PAGE might therefore result from alternative phosphorylation, from other post-translational modifications or from cryptic cleavages within the SP1 sequence. The Gag subdomain located between MA and CA of different retroviruses differ in many aspects and have a wide range of functions [25,45-47]. In MPMV, RSV and MLV they encode viral late domains and there is even a PTAP motive at the C-terminus of the HERV-K(HML-2) p15 protein. The presence of a substantial quantity of the 36 kDa MA-SP1-p15 intermediate in our VLP preparations suggests that the cleavage at the p15-CA junction occurs at a much higher rate than the liberation of mature MA and p15 domains. The processing of the unconventional SP1-p15 site particularly appears to proceed at a slow rate. We hypothesize that to a very substantial degree, SP1 and p15 remain uncleaved from the MA subdomain after the mature core of a HERV-K(HML-2) particle has already formed.

## Conclusion

A prerequisite for the infectivity of retroviral particles is a maturation process in which Gag precursor proteins are cleaved at precise positions by the viral PR. In all orthoretroviruses, proteolytic processing generates the MA, CA, and NC proteins. Depending on the viral genus and species several additional proteins and peptides are released. To characterize the Gag cleavage of HERV-K(HML-2), we have recovered the original sequence of the prototypical HERV-K113 element by reversing post-insertional mutations acquired by the provirus during chromosomal residency and have facilitated the expression and production of maturation-competent VLPs by partial codon optimization of the *gag-pro-pol *region. The characterization of the liberated mature Gag proteins and cleavage sites of HERV-K(HML-2) will facilitate molecular cloning, allow a deeper analysis of these proteins and should promote further research into the maturation process of betaretroviruses. Studies of retroviral evolution and phylogeny will also benefit from a comparative analysis of the maturation characteristics of this ancient virus and contemporary retroviruses. Our results might also be of help in determining the underlying reasons for the structural and functional handicaps of HERV-K(HML-2) particles expressed in human tumours. Since HERV-K(HML-2) expression is associated with a variety of cancers and autoimmune diseases, a detailed knowledge of the fundamental viral characteristics may help us elucidate the molecular and potentially pathogenic nature of this association.

## Materials and methods

### Cell culture

HEK 293T cells were grown in complete Dulbecco's modified Eagle medium containing 10% fetal bovine serum, penicillin (50 U/ml), streptomycin (50 μg/ml) and L-glutamine (2 mM).

### DNA synthesis, cloning and mutagenesis

Codon-optimization and production of synthetic HERV-K(HML-2) sequences was conducted by the company GeneArt (Regensburg, Germany) and has been described previously [19]. The synthetic partially codon-optimized *gag-pro-pol *sequence was cloned into the pcDNA3.1 vector using the Kpn I and Xho I restriction sites to obtain the pcDNAoricoHERV-K_GagProPol construct. Mutations to inactivate the PR and to substitute amino acids adjacent to the cleavage site were performed using the QuikChange Multi Site-Directed Mutagenesis Kit (Stratagene).

### Concentration of VLPs

4 × 10^6 ^HEK 293T cells were seeded into 100 mm dishes and one day later transfected with 20 μg plasmid DNA using the calcium phosphate method. 48 hours post-transfection the supernatants were collected, centrifuged at 3345 × g for 8 min and filtered through 0.45 μm-pore-size membranes to remove cell debris. The viral particles were then concentrated by ultracentrifugation through a 20% sucrose cushion at 175,000 × g for 3 h at 4°C. Viral pellets were resuspended in either 100 μl 5 M urea containing 1% glacial acidic acid for high pressure liquid chromatography or in 0.05 M Hepes buffer, pH 7.2 for Western blot analysis.

### Electron microscopy

Two days post-transfection, cells were fixed with 2.5% glutaraldehyde in 0.05 M Hepes (pH 7.2) for 1 h at room temperature before harvesting by scraping and centrifugation (2000 × g). Cell pellets were post-fixed with OsO4 (1% in ddH2O; Plano, Wetzlar, Germany), block-stained with uranyl acetate (2% in ddH2O; Merck, Darmstadt, Germany), dehydrated stepwise in graded alcohol, immersed in propylenoxide and embedded in Epon (Serva, Heidelberg) with polymerisation at 60°C for 48 h. Ultrathin sections (60-80 nm) were cut using an ultramicrotome (Ultracut S or UCT; Leica, Germany) and stained with 2% uranyl acetate and lead citrate. Transmission electron microscopy was performed with an EM 902 (Zeiss) operated at 80 kV and the images were digitised using a slow-scan charge-coupled-device camera (Pro Scan; Scheuring, Germany).

For SEM, the transfected cells were immersed overnight in 2.5% glutaraldehyde (in 0.05 M Hepes buffer, pH 7.2) and gently washed with distilled water prior to postfixation (1% OsO_4_, 1 h). Samples were then rinsed with distilled water, dehydrated with alcohol (30-96%), critical point dried and sputter-coated with 7 nm gold-palladium (Polaron Sputter Coating Unit E 5100, GaLa Instrumente, Bad Schwalbach). The samples were examined using a LEO 1530 scanning electron microscope (Carl Zeiss SMT AG, Oberkochen) operated at 3 kV.

### Reversed phase high pressure liquid chromatography

Protein separation was performed on an Agilent (Palo Alto, CA) 1200 series binary HPLC fitted with a 4.6 × 150 mm 3.5 micron Zorbax 300SB-C8 reverse-phase column (Agilent, Palo Alto, CA). The column was maintained at 40°C. Solvent A consisted of 0.1% trifluoroacetic acid in water and solvent B of 0.08% trifluoroacetic acid in acetonitrile. Proteins were eluted at a flow rate of 0.5 ml/min from 8-58 min and 1 ml/min from 0-6 min and 60-62 min employing the following gradient: solvent B, 0-6 min, 0%; 8 min, 15%; 49 min, 40%; 58 min, 95%; 58-60 min, 95%; 62 min, 0%. The eluate was monitored at 280 nm and 0.5 ml fractions were collected.

### Sample preparation for MALDI-TOF mass spectrometry

Following evaporation to dryness proteins of each HPLC fraction were dissolved in 20 μl of TA2 (2:1 (v/v) mixture of 100% acetonitrile and 0.3% TFA). 1 μl of each fraction was spotted onto a 384-spot polished steel target plate (Bruker Daltonics, Bremen, Germany) and mixed with 1 μl alpha-Cyano-4-hydroxy-cinnamic acid (HCCA) solution (6 mg/ml in TA2) and air dried.

### Parameters of MALDI-TOF mass spectrometry

Mass spectra were collected by an Autoflex I mass spectrometer (Bruker Daltonics). The instrument was controlled by Bruker's FlexControl 3.0 data collection software and was equipped with a UV-nitrogen laser (λ = 337 nm). MS measurements were carried out in linear mode using an acceleration voltage of 20.00, or 18.45 kV (ion source 1 and 2), respectively. Lens voltage was 6.70 kV. Spectra were stored in mass range between 0.7-10 kDa and 2-20 kDa, depending on the expected size of the peptides. External calibration was performed employing protein calibration standard I and peptide calibration standard II, respectively (Bruker Daltonics). To achieve a high signal to noise ratio each spectrum represents the integration of at least 600 individual laser shots. In order to determine the exact positions of the N- and C-terminal ends of the processed Gag fragments the respective proteins were digested with trypsin. Tryptic peptides were purified using ZipTip C18 tips (Millpore, Bedford, MA, USA) and measured in the reflectron mode using an acceleration voltage of 19.40, or 16.90 kV (ion source 1 and 2), respectively. Lens voltage was 8 kV. Spectra were stored in the mass range between 0.7-4 kDa. Mass peaks that did not correspond to tryptic peptides predicted by theoretical *in silico *tryptic digests were further analysed by MS/MS to generate sequence information.

### *De novo *protein sequencing

Sequencing by MALDI-TOF MS was carried out under the control of FlexControl software (Bruker Daltonics) using an Ultraflex II MALDI-TOF/TOF mass spectrometer (Bruker Daltonics) equipped with a near infrared solid state smartbeam™ laser Nd:YAG laser (λ = 1064 nm) which operated at 100 Hz. Fractions containing target peptides were identified by recording spectra in linear positive mode with external calibration using a standard mixture of peptides. In order to assign a mass window for fragmentation and peptide sequencing in the 'LIFT' MS/MS mode, an exploratory scan from 2000 to 5000 Da was performed in the reflectron mode. Spectra were obtained by averaging up to 3000 laser shots acquired at a fixed laser power, which had been set to the minimum laser power necessary for ionization of selected samples before starting the analyses. The mass spectra were visualized and processed using FlexAnalysis software and sequence tag hints were obtained by analyzing tandem MS spectra employing the Biotools 3.0 software (Bruker Daltonics). For N-terminal sequencing by Edman degradation, proteins were separated by sodium dodecyl sulphate-polyacrylamide gel electrophoresis (SDS-PAGE), blotted to a PVDF membrane and stained with Ponceau S. Protein bands of the expected size were cut out and sent for sequencing (Proteome Factory, Berlin, Germany).

### Immunization

Alignments of several Gag proteins of betaretroviruses closely related to HERV-K113 provided clues to the putative Gag subdomains (data not shown). Accordingly, three fragments with sequences corresponding to the putative MA subdomain (amino acids 1-100), the putative p15 subdomain (140-282) and the putative CA subdomain (283-526) were generated. Each fragment was inserted into the pET16b vector (Novagen, Gibbstown, USA), expressed in BL21 *E. coli *and affinity purified on a Ni-NTA column. Proteins were eluted in 8 M urea and dialyzed against phosphate buffered saline (PBS). 100 μg of recombinant Gag-protein fragments were then used for immunization of Wistar rats and sera collected throughout the period of four immunizations. These animal experiments were performed according to institutional and state guidelines.

### SDS gel, silver nitrate staining and Western blot analysis

Virus particles were mixed with Laemmli sample buffer (Bio-Rad), briefly boiled and subjected to SDS-PAGE. The proteins were then visualized by silver nitrate staining using the Bio-Rad Silver Stain Kit or blotted using the semidry transfer method to a PVDF membrane (Roth). After transfer, blots were blocked in blocking buffer (PBS, 5% skim milk powder, 0.1% Tween) and incubated with the appropriate primary antibodies. Visualization of the proteins was achieved using secondary antibodies coupled to horseradish peroxidise, enhanced chemiluminescence reagents and autoradiographic film.

## Competing interests

The authors declare that they have no competing interests.

## Authors' contributions

NBa, RK and MG conceived and drafted the study. MG planned and coordinated the experiments and performed the virus purification, mutagenesis, Western blot analysis as well as participated in the HPLC and MS experiments. TS and DN planned and performed the HPLC and MS experiments. NBa and NBe reconstructed the original sequence of HERV-K113 (oriHERV-K113) via sequence alignment and mutagenesis. NBe, MG, CC and AZ carried out sequence alignments and mutagenesis reactions. OH performed the protein purification, immunization and characterization of the rat sera. The work was supported in part by a donation from the Heinz Kuthe de Mouson legacy to R.K. All authors read and approved the final manuscript.

## Supplementary Material

Additional file 1 **Identification of post-insertional amino acid substitutions in the Gag-Pro-Pol region of HERV-K113**. (A) The amino acid sequences of HERV-K101, HERV-K102, HERV-K104, HERV-K107, HERV-K108, HERV-K109, HERV-K115, AP000776 and AC025420 and Y17833 were aligned to the sequence of HERV-K113. The consensus amino acid sequence and a sequence allowing some degree of shared polymorphism (oriHERV-K113) were deduced from the alignments (see results for details). The oriHERV-K113 sequence is assumed to represent the original proteins of the virus on the day of integration. The positions of the Gag cleavage sites identified in this report are indicated by arrowheads. (B) To enhance expression, a partially codon-optimized sequence was generated. The codon-optimized regions are highlighted in yellow. Click here for file
