# Supplementary material for: Identification of the protease cleavage sites in a reconstituted Gag polyprotein of an HERV-K(HML-2) element
Source: Retrovirology. 2011 May 9;8:30. doi: 10.1186/1742-4690-8-30 (PMC3114732; doi:10.1186/1742-4690-8-30)
Supplement: Additional file 1 — Identification of post-insertional amino acid substitutions in the Gag-Pro-Pol region of HERV-K113. (A) The amino acid sequences of HERV-K101, HERV-K102, HERV-K104, HERV-K107, HERV-K108, HERV-K109, HERV-K115, AP000776 and AC025420 and Y17833 were aligned to the sequence of HERV-K113. The consensus amino acid sequence and a sequence allowing some degree of shared polymorphism (oriHERV-K113) were deduced from the alignments (see results for details). The oriHERV-K113 sequence is assumed to represent the original proteins of the virus on the day of integration. The positions of the Gag cleavage sites identified in this report are indicated by arrowheads. (B) To enhance expression, a partially codon-optimized sequence was generated. The codon-optimized regions are highlighted in yellow. [file 1742-4690-8-30-S1.PDF]

Additional File 1A

Alignment of Gag

```
                    10         20         30         40         50         60         70         80         90        100
            ....|....| ....|....| ....|....| ....|....| ....|....| ....|....| ....|....| ....|....| ....|....| ....|....|
HERV-K113   MGQTKSKIKS KYASYLSFIK ILLKRGGVKV STKNLIKLFQ IIEQFCPWFP EQGTLDLKDW KRIGKELKQA GRKGNIIPLT VWNDWAIIKA ALEPFQTEED
HERV-K101   .......... .......... .......... .......... .......... .......... .......... .......... .......... ..........
HERV-K102   .......... .......... .......... .......... .......... .......... .......... .......... .......... ..........
HERV-K104   .......... .......... .......... .......... .......... .......... .......... .......... .......... ..........
HERV-K107   .......... .......... .......... .......... .......... ....S..... .......... .......... .......... ..........
HERV-K108   .......... .......... .......... .......... .......... .......... .......... .......... .......... ..........
HERV-K109   .......... .......... .......... .......... .......... .......... .......... .......... .......... ..........
HERV-K115   .......... .......... .......... .......... .......... .......... .......... .......... .......... ..........
AP00076     .......... .......... .......... .......... .......... .......... .......... .......... .......... ..........
AC025420    .......... .......... .......... .......... .......... .......... .......... .......... .......... ..........
Y17833      .......... .......... .......... .......... .......... .......... .......... .......... .......... ..........
Consensus   .......... .......... .......... .......... .......... .......... .......... .......... .......... ..........
oriHERV-K113 .......... .......... .......... .......... .......... .......... .......... .......... .......... ..........

                   110        120        130        140        150        160        170        180        190        200
            ....|....| ....|....| ....|....| ....|....| ....|....| ....|....| ....|....| ....|....| ....|....| ....|....|
HERV-K113   SVSVSDAPGS CIIDCNEKTR KKSQKETESL HCEYVAEPVM AQSTQNADYN QLQEVIYPET LKLEGKGPEL MGPSESKPRG TSPLPAGQVP VTLQPQKQVK
HERV-K101   .......... .......... .......... .......... ......V... .......... .......... V......... .......... ..........
HERV-K102   .......L.. .......N.. ........G. .......... ......V... .......... .......... V......... ..H....... ..........
HERV-K104   .......... .L........ ......M.G. .......... ......V... .......... .......... V.L....*.. .......... ......T...
HERV-K107   .I........ .L.....N.. .......... .......... ......V... .......... .......... .......... .........L .R........
HERV-K108   .......... .......N.. ........G. .......... ......V... .......... .......... V......... .......... ..........
HERV-K109   .I........ G......... .......... .......... ......V... .......... .......... V......... .......... ..........
HERV-K115   .I........ .L.....N.. .......... .......... ......V... .......... .......... V......... .......... ..........
AP00076     .I........ .L........ ........G. .......... ......V... .......... .......... V......*.. .......... .R....T...
AC025420    .I........ .......N.. ........G. ....A..... ......V... .......... .......... V......... .......... ......T...
Y17833      .......... .......N.. .......... .......... ......V... .......... .......... V......... ..R....... ......T...
Consensus   .......... .......N.. .......... .......... ......V... .......... .......... V......... .......... ..........
oriHERV-K113 .......... .......... .......... .......... ......V... .......... .......... V......... .......... ..........

                   210        220        230        240        250        260        270        280        290        300
            ....|....| ....|....| ....|....| ....|....| ....|....| ....|....| ....|....| ....|....| ....|....| ....|....|
HERV-K113   ENKTQPPVAY QYWPPAELQY QPPPESQYGY PGMPPAPQGR APYPQPPTRR LNPTAPPSRQ GSELHEIIDK SRKEGDTEAW QFPVTLELMP PGEGAQEGEP
HERV-K101   .......... .......... R......... .......... .......... .......... .......... .......... .......P.. ..........
HERV-K102   .......... .......... R......... .......... .......... .......... .......... .......... .......P.. ..........
HERV-K104   .......... ...L...... R......... ..I....... V......... ...M...... .......... .......... .......P.. ..........
HERV-K107   ......Q... ....L..... R......... .......... ...H...... ...M...... .......... .......... .......P.. ..........
HERV-K108   .......... .......... R......... .......... .......... .......... ..K....... .......... .......P.. ..........
HERV-K109   .......... .......... R......... .......... .......... .......... .......... .......... .......P.. ..........
HERV-K115   .......... .......... R......... .......... E......... .......... .......... .......... .......P.. ..........
AP00076     .......... .......... R......... .......... .......... .......... .......... .......... .......PK. ..........
AC025420    .......... .......... R......... .......... .......... .......... .......... .......... ....M..P.. ..........
Y17833      .......... .......... R..V...... .......... .......... .........R .......... .......... ....M..P.. ..........
Consensus   .......... .......... R......... .......... .......... .......... .......... .......... .......P.. ..........
oriHERV-K113 .......... .......... R......... .......... .......... .......... .......... .......... .......P.. ..........

                   310        320        330        340        350        360        370        380        390        400
            ....|....| ....|....| ....|....| ....|....| ....|....| ....|....| ....|....| ....|....| ....|....| ....|....|
HERV-K113   PTVEARYKSF SIKMLKDMKE GVKQYGPNSP YMRTLLDSIA HGHRLIPYDW EILAKSSLSP SQFLQFKTWW IDGVQEQVRR NRAANPPVNI DADQLLGIGQ
HERV-K101   .......... .......... .......... .......... Y......... .......... .......... .......... .......... ..........
HERV-K102   .......... .......... .......... .......... .......... .......... .......... .......... .......... ..........
HERV-K104   L......... .......... .......... .......... .......... .......... .L........ .......... .......... .......T..
HERV-K107   .......... .......... .......... .......... Y......... .......... .......... .......... .......... ..........
HERV-K108   .......... ...K...... .......... .......... .......... .......... .......... .......... .......... ..........
HERV-K109   .......... ...I...... .......... .......... .......... .......... .......... .......... .......... ..........
HERV-K115   .......... .......... .......... .......... .......... .......... .......... .......... .......... ..........
AP00076     .......... .......... .......... .......... .......... .......... .......... .......... .......... ..........
AC025420    .......... .......... .......... .......... .......... ........L. .......... ........Q. .......... ..........
Y17833      .......... .......... .......... .......... .......... .......... .......... .......... .......... ..........
Consensus   .......... .......... .......... .......... .......... .......... .......... .......... .......... ..........
oriHERV-K113 .......... .......... .......... .......... .......... .......... .......... .......... .......... ..........

                   410        420        430        440        450        460        470        480        490        500
            ....|....| ....|....| ....|....| ....|....| ....|....| ....|....| ....|....| ....|....| ....|....| ....|....|
HERV-K113   NWSTISQQAL MQNEAIEQVR AICLRAWEKI QDPGSTCPSF NTVRQGSKEP YPDFVARLQD VAQKSIADEK ARKVIVELMA YENANPECQS AIKPLKGKVP
HERV-K101   .......... .......... .......... .....A.... .......... .......... .......... .......... .......... ..........
HERV-K102   .......... .......... .......... .......... .......... .......... .......... .......... ......-... ..........
HERV-K104   .......... .......... .......... .....A.... .......... .L........ .......N.. .H........ .....L.... ......A...
HERV-K107   .......... .......... .......... .......... .......... .......... .......... .G........ .......... ..........
HERV-K108   .......... .......... .......... .......... .......... .......... .......... .......... .......... ..........
HERV-K109   .......... .......... .......... .......... .......... .......... .......... .......... .......... ..........
HERV-K115   .......... .......... .......... .......... .......... .......... .......... .......... .......... ..........
AP00076     .......... .......... .......... .......... .......... .......... .......... .......... .......... ..........
AC025420    .......... .......... .......... .......... .....S.... .......... .......... .......... .......... ..........
Y17833      .......... .......... .......... .......... .......... .......... .......N.. .......... ...P...... ..........
Consensus   .......... .......... .......... .......... .......... .......... .......... .......... .......... ..........
oriHERV-K113 .......... .......... .......... .......... .......... .......... .......... .......... .......... ..........
```

```
                   510        520        530        540        550        560        570        580        590        600
              ....|....| ....|....| ....|....| ..▼.|....| ....|....| ....|....| ....|....| ....|....| ....|....| ....|....|
HERV-K113     AGSDVISEYV KACDGMGGAM HKAMLMAQAI TGVVLGGQVR TFGGKCYNCG QIGHLKKNCP VLNKQNITIQ ATTTGREPPD LCPRCKKGKH WASQCRSKFD
HERV-K101     .......... .....I.... .......... .......... .......... .......... .......... .......... .......... ..........
HERV-K102     .......... .....I.... .......... .......... .......... .......... .......... .......... .......... ..........
HERV-K104     .......... .....I.... .......... .......... .......... .......... .......... ........A. .......... .....C....
HERV-K107     .......... .....I.... .......... .......... .......... .......... .......... .......... .......... ..........
HERV-K108     .......... .....I.... .......... .......... ...R...... .......... .......... .......... .......... ..........
HERV-K109     .......... .....I.... .......... .......... .......... .......... .......... .......V.. .......... ..........
HERV-K115     .......... .....I.... .......... .......... .......... .......... .......... .......... .......... ..........
AP00076       .......... .....I.... .......... .......... .......... .......... .......... .......... .......... ..........
AC025420      .......... .....I.... .......... .......... .......... .......... .......... .......... .......... ..........
Y17833        .......... .......... .......... .......... .......... .......... .......... .......... .......... ..........
Consensus     .......... .....I.... .......... .......... .......... .......... .......... .......... .......... ..........
oriHERV-K113  .......... .....I.... .......... .......... .......... .......... .......... .......... .......... ..........

                   610        620        630        640        650        660
              ....|....| ....|....| ....▼....| ....|....| ....|.▼..| ....|....| ....|..
HERV-K113     KNGQPLSGNE QRGQPQAPQQ TGAFPIQPFV PQGFQGQQPP LSQVFQGISQ LPQYNNCPPP QAAVQQ*
HERV-K101     .......... .......... .......... .......... .......... ........L. ......*
HERV-K102     .......... .......... .......... .....E.... .......... .......... ......*
HERV-K104     .......... .......... .......... .......... .......... .......... ......*
HERV-K107     .......... .......... .......... .......... .......... ........S. ......*
HERV-K108     .......... .......... .......... .......... .......... .......... ......*
HERV-K109     .......... .......... .......... .......... .......... .......... .V....*
HERV-K115     .......... .......... .......... ....-..... .......... .......... ......*
AP00076       .......... .......... .......... .......... .......... .......... ......*
AC025420      .......... .......... .......... .......... .......... .......... ......*
Y17833        .......... .......... .......... .H........ .......... .......... ......*
Consensus     .......... .......... .......... .......... .......... .......... ......*
oriHERV-K113  .......... .......... .......... .......... .......... .......... ......*
```

Alignment of Pro

```
                      10         20         30         40         50         60         70         80         90        100
              ....|....| ....|....| ....|....| ....|....| ....|....| ....|....| ....|....| ....|....| ....|....| ....|....|
HERV-K113     KWATIVGKRA KGPASGPTTN WGIPNSAICS SGFSGTTTPT VPSVSGNKPV TTIQQLSPAT SGSAAVDLCT IQAVSLLPGE PPQKIPTGVY GPLPEGTVGL
HERV-K101     .......... .......... .......... .......... .......... .......... .S........ .......... .......... ..........
HERV-K102     .......... .......... .......... .......... .......... .......... .......... .......... .......... ..........
HERV-K104     ........*. .......... .......... .......... .......... .......... .......... .......... .......... ..........
HERV-K107     .......... .......... .......... .......... .......... .......LT. .......... .......... .......... ..........
HERV-K108     .......... .......... .......... .......... .......... .......... .......... .......... ....T..... ....K.....
HERV-K109     .......... .......... .......... .......... .......... .......... .......... .......... .......... ..........
HERV-K115     .......... .......... .......... ....-..... .......... .......... .......... .......... .......... ..........
AP00076       .......... .......... .......... .......... .......... .......... .......... .......... .......... ..........
AC025420      .......... .......... .......... .......... .......... ........T. .......... .......... .......... ..........
Y17833        .......... .......... .......... .W........ .......... .......... .......... .......... .......... ..........
Consensus     .......... .......... .......... .......... .......... .......... .......... .......... .......... ..........
oriHERV-K113  .......... .......... .......... .......... .......... .......... .......... .......... .......... ..........

                     110        120        130        140        150        160        170        180        190        200
              ....|....| ....|....| ....|....| ....|....| ....|....| ....|....| ....|....| ....|....| ....|....| ....|....|
HERV-K113     ILGRSSLNLK GVQIHTSVVD SDYKGEIQLV ISSSIPWSAS PGDRIAQLLL LPYIKGGNSE IKRTGGLGST DPTGKAAYWA SQVSENRPVC KAIIQGKQFE
HERV-K101     .......... .......... .......... .......... ....S..... .......... L.KI...... .......... .......... ..........
HERV-K102     .......... .......... .......... .......... .......... .......... ...I...... .......... ...T...... ..........
HERV-K104     .......... .......... T......... ....V..... .......... .......... ...I.EF... .......... .......... ..........
HERV-K107     .......... .......... .......... .......... .R........ .......... ...I...V.. .......... .......... ..........
HERV-K108     .......... .......... .......... .......... .R........ .......... ...I...... .......... .......... ..........
HERV-K109     .......... .......... .......... ....V..... .......... .......... ...I...... .......... .......... ..........
HERV-K115     .......... .......... .......... .......... .R........ .......... ...I...... .......... .......... ..........
AP00076       .......... .......... .....K.... .........I .......... .......... ...I...V.. .......... .......... ..........
AC025420      .......... .......... .......... .......... .......... .......... ..II...... .......... .......... ..........
Y17833        .......... ......G... .......... .......... .......... .......... ...I...... .......... .......... ..........
Consensus     .......... .......... .......... .......... .......... .......... ...I...... .......... .......... ..........
oriHERV-K113  .......... .......... .......... .......... .......... .......... ...I...... .......... .......... ..........

                     210        220        230        240        250        260        270        280        290        300
              ....|....| ....|....| ....|....| ....|....| ....|....| ....|....| ....|....| ....|....| ....|....| ....|....|
HERV-K113     GLVDTGADVS IIALNQWPKN WPKQKAVTGL VGISTASEVY QSTEILHCLG PDNQESTVQP MITSIPLNLW GRDLLQQWGV EITMPAPLYS PTSQKIMTKM
HERV-K101     .......... .......... .......... ...G...... .......... .......... .......... .........A .......... ..........
HERV-K102     .......... .......... .......... ...G...... .......... .......... .......... .........A .......... ..........
HERV-K104     .....E.... .......... .......... ...G...... .......... .......... .......... .Q.......A .......... ..........
HERV-K107     .......... .......... .......... ...G...... ..M....... .......... .......... .........A .......... ..........
HERV-K108     .......... .......... .......... ...G...... .......... .......... .......... .........A .......S.. ..........
HERV-K109     .......... .......... .......... ...G...... ..M....... .......... .......... .........A .......... .........R
HERV-K115     .......... .......... .......I.. ...G...... ..M....... .......... .......... .........A .......... .........R
AP00076       .......... .......... .......... ..VG...... .......... .......... .......... .........A .......... ..........
AC025420      .......... .......... .......... ...G...... .......... .......... .......... .........A .....T.... ..........
Y17833        .......... .......... .......... ...G...... .......... .......... .......... .........A .......... ..........
Consensus     .......... .......... .......... ...G...... .......... .......... .......... .........A .......... ..........
oriHERV-K113  .......... .......... .......... ...G...... .......... .......... .......... .........A .......... ..........
```

```
                     310        320        330
                 ....|....| ....|....| ....|....| ....|
HERV-K113        GYIPGKGLGK NEDGIKVPVE AKINQEREGI GYPF*
HERV-K101        .......... ......I.F. .....K.... ....*
HERV-K102        .......... .......... .......... ....*
HERV-K104        .......... .......... .....K.... ....*
HERV-K107        .......... .......... .......... ....*
HERV-K108        .......... ......I... .......... .N.C*
HERV-K109        .......... ......I.F. .....K.... ....*
HERV-K115        .......... ......I.F. .....K.... ....*
AP00076          .......... ......I... .....K.... ....*
AC025420         .......... .......... .....K.... ....*
Y17833           ...L...... ......I... .....K.... ....*
Consensus        .......... ......I... .....K.... ....*
oriHERV-K113     .......... .......... .......... ....*
```

Alignment of Pol

```
                          10         20         30         40         50         60         70         80         90        100
                 ....|....| ....|....| ....|....| ....|....| ....|....| ....|....| ....|....| ....|....| ....|....| ....|....|
HERV-K113        NKSRKRRNRV SFLGAATVEP PKPIPLTWKT EKPVWVNQWP LPKQKLEALH LLANEQLEKG HIEPSFSPWN SPVFVIQKKS GKWRMLTDLR AVNAVIQPMG
HERV-K101        ...K...... .......I.. .......... .........Q .......... .......... .......... .......... .......... ..........
HERV-K102        .......... .......... .......... .......... .......... .......... .......... .......... .......... ..........
HERV-K104        ...K...... .......... .......... .......... .......... .......... .......... .......... .......... ....I.....
HERV_K107        .......... .....V.... .......... .......... .......... .......... .......... .......... ...HT..... ..........
HERV-K108        .........E .L........ .......... .......... .......... .......... .......... .......... .......... ..........
HERV-K109        ...K...... .......I.. .......... .......... .......... .......... .......... .......... .......... ..........
HERV-K115        ...K...... ....V..I.. .......... ..L....... .......... .......... .......... .......... .......... ..........
AP00076          ...K...... .......... .......... .......... .......... .......... .......... .......... .......... ..........
AC025420         ...K...... .......L.. .......... ..L....... .......... .......... .......... F......... .......... ....I.....
Y17833           ...K...... .......... .......... .......... .......... .......... .......... .......... ..........V..........
Consensus        ...K...... .......... .......... .......... .......... .......... .......... .......... .......... ..........
oriHERV-K113     .......... .......... .......... .......... .......... .......... .......... .......... .......... ..........

                         110        120        130        140        150        160        170        180        190        200
                 ....|....| ....|....| ....|....| ....|....| ....|....| ....|....| ....|....| ....|....| ....|....| ....|....|
HERV-K113        PLQPGLPSPA MIPKDWPLII IDLKDCFFTI PLAEQDCEKF AFTIPAINNK EPATRFQWKV LPQGMLNSPT ICQTFVGRAL QPVRDKFSDC YIIHYIDDIL
HERV-K101        .......... .......... .......... .......... .......... .......... .......... .......... ....E..... ..........
HERV-K102        .......... .......... .......... .......... .......... .......... .......... .......*.. ....E..... ..........
HERV-K104        ...SR..... .......... .......... .......... ...VL..... .......... .......... .......... ....E..... ..........
HERV_K107        .......... .......... .......... .......... .......... .......... .......... .......... ....E..... ..........
HERV-K108        .......... .......... .......... .......... .......... .......... .......... .......... ....E..... ....C.....
HERV-K109        .......... .......... .......... .......... .......... .......... .......... .......... ....-..... ..........
HERV-K115        .......... .......... .......... .......... .......... .......... .......... .......... ....K..... ..........
AP00076          .......... .......... .......... .......... .......... .......... .......... .......... ....E..... ..........
AC025420         .......... .......... .......... .......... .......... .......... .......... .......... ....E..... ..........
Y17833           ........L. .......... .......... .......... .......... .......... .......... .......... ....E..... ..........
Consensus        .......... .......... .......... .......... .......... .......... .......... .......... ....E..... ..........
oriHERV-K113     .......... .......... .......... .......... .......... .......... .......... .......... ....E..... ..........

                         210        220        230        240        250        260        270        280        290        300
                 ....|....| ....|....| ....|....| ....|....| ....|....| ....|....| ....|....| ....|....| ....|....| ....|....|
HERV-K113        CAAETKDKLI DCYTFLQAEV ANAGLAIASD KIQTSTPFHY LGMQIENRKI KPQKIEIRKD TLKTLNDFQK LLGDINWIRP TLGIPTYVMS NLFSILRGDS
HERV-K101        .......... .......... .S........ .......... .........- .......... .......... .......... .......A.. ..........
HERV-K102        .....R.... .......... .......... .......... .......... ....V..... .......... .......... .......A.. ..........
HERV-K104        .......... .......... .......... .......... .......... .........Y .......... .......... .......A.. ..........
HERV_K107        .......... .......... .......... .......... .......... .......... .......... .......... .......A.. ..........
HERV-K108        .......... .......... .......... .......... .......... .......... .......... .......... .......A.. ..........
HERV-K109        .......... .......... .......... .......... .......... .......... .......... ........W. .......A.. ..........
HERV-K115        .......... .......... .S........ .......... .......... .......... .......... ........Q. .......A.. ..........
AP00076          .......... .......... .......... .......... .......... .......... ...A...... .......... .......A.. ..........
AC025420         .......... .......... .......... .......... ..I....... -......... .......... .......... .......A.. ..........
Y17833           ....M..... .......... .......... .......... .E........ ..P....... .......... .......... .......A.. ..........
Consensus        .......... .......... .......... .......... .......... .......... .......... .......... .......A.. ..........
oriHERV-K113     .......... .......... .......... .......... .......... .......... .......... .......... .......A.. ..........

                         310        320        330        340        350        360        370        380        390        400
                 ....|....| ....|....| ....|....| ....|....| ....|....| ....|....| ....|....| ....|....| ....|....| ....|....|
HERV-K113        DLNSKRMLTP ETTKEIKLVE EKIQSAQINR IDPLAPLRLL IFATAHSPIG IIIQNTDLVE WSFLPHSTVK TFTLYLDQIA TLIGQTRLRI IKLCGNDPDK
HERV-K101        ......I... .A........ .......... ....T..Q.. ........T. .......... .......... ........M. .......... .......Q..
HERV-K102        ......I... .A........ .......... .......Q.. ........T. .......... .......... .......... .......... ...Y......
HERV-K104        .......... .A........ .....V.... .......Q.. ........T. .......... .......... .......... .......... ..........
HERV_K107        ....Q.I... .A........ .......... .......Q.. ........T. .......... .......... .......... .......... T.........
HERV-K108        .......... .A........ .......... .......Q.. ........T. .......... .......... .......... .......... ..........
HERV-K109        .......... .A........ .......... .......Q.. ........T. .......... .......... .......... .......... ..........
HERV-K115        ......I... .A........ .......... .......Q.. ........T. .......... .......... .......... .......... ..........
AP00076          .......... .A........ .......... .......Q.. ........T. .......... .......... .......... .......... ..........
AC025420         ...----... .A........ .......... ......-Q.. ........T. .......... .......... .......... .......... ..........
Y17833           .......... .A........ .......... .......Q.. ........T. .......... .......... ........M. .......... ..........
Consensus        .......... .A........ .......... .......Q.. ........T. .......... .......... .......... .......... ..........
oriHERV-K113     .......... .A........ .......... .......Q.. ........T. .......... .......... .......... .......... ..........
```

```
                      410        420        430        440        450        460        470        480        490        500
                ....|....| ....|....| ....|....| ....|....| ....|....| ....|....| ....|....| ....|....| ....|....| ....|....|
HERV-K113       IVVPLTKEQV RQAFINSGAW QIGLANFVGI IDNHYPKTKI FQFLKLTTWI LPKITRREPL ENALTVFTDG SSNGKAAYTG LKERVIKTPY QSAQRAELVA
HERV-K101       .......... .......... .......... .......... .......... .......... .......... .......... P......... ..........
HERV-K102       .......... .......... .......... .......... .......... .......... .......... .......... P......... ..........
HERV-K104       .......... ..D....... .......... .......... .......... ......H... .......... .......... P..*...... ..........
HERV_K107       .......... .......... .........L .......... .......... .......... .......... .......... P......... ..........
HERV-K108       .......... .......... K......... .......... .......... .......... .......... .......... P......... ..........
HERV-K109       .......... .......... K......... .......... .......... .......... .......... .......... P......... ..........
HERV-K115       .......... .......... .......... .......... .......... .......... .......... .......... P......... ..........
AP00076         .......... .......... .......... .......... .......... .......... .......... .......... P......... ..........
AC025420        .......... .......... .......... .......... .......... .......... .......... .......... P......... ..........
Y17833          .......... .......... .......... .......... .....M.... .......... .......... .......... P.......Q. ..........
Consensus       .......... .......... .......... .......... .......... .......... .......... .......... P......... ..........
oriHERV-K113    .......... .......... .......... .......... .......... .......... .......... .......... P......... ..........

                      510        520        530        540        550        560        570        580        590        600
                ....|....| ....|....| ....|....| ....|....| ....|....| ....|....| ....|....| ....|....| ....|....| ....|....|
HERV-K113       VITVLQDFDQ PINIISDSAY VVQATRDVET ALIKYSMDDQ LNQLFNLLQQ TVRKRNFPFY ITHIRAHTNL PGPLTKANEQ ADLLVSSALI KAQELHALTH
HERV-K101       .......... .......... .......... .......... .......... .......... .......... .......... .......... ..........
HERV-K102       .......... .......... .......... .......... .......... .......... .......... .......... .......... ..........
HERV-K104       .......... .......... .......... .......... .......... .......... .......... .......... ........F. ..........
HERV_K107       .......... .......... .......... .......... .......... .......... .......... .......... .......... ..........
HERV-K108       .......... .......... .......... .......... .......... .......... .......... .......... .......... ..........
HERV-K109       .......... .......... .......... .......... .......... .......... .......... .......... .......... ..........
HERV-K115       .......... .......... ......V... .......... .......... .......... .......... .......... .......... ..........
AP00076         ........-- -......... .......... .......... .......... .......... .......... ........K. .......... ..........
AC025420        .......... ..S....... .......... .......... .......... .......... .......... .......... V...A..... ..........
Y17833          .......... .......... .......... .......... .......... .......... .......... .......... .......... ..........
Consensus       .......... .......... .......... .......... .......... .......... .......... .......... .......... ..........
oriHERV-K113    .......... .......... .......... .......... .......... .......... .......... .......... .......... ..........

                      610        620        630        640        650        660        670        680        690        700
                ....|....| ....|....| ....|....| ....|....| ....|....| ....|....| ....|....| ....|....| ....|....| ....|....|
HERV-K113       VNAAGLKNKF DVTWKQAKDI VQHCTQCQVL HLPTQEAGVN PRGLCPNALW QMDVTHVPSF GRLSYVHVTV DTYSHFIWAT CQTGESTSHV KKHLLSCFAV
HERV-K101       .......... .......... .......... .......... .......... .......... .......... .......... .......... ..........
HERV-K102       .......... .......... ........I. .......... .......... .......... .......... .......... .......... ..........
HERV-K104       .......... .......... .......... .......... .......... .......... .......... .......... .......... ..........
HERV_K107       .......... .......... .......... .......... .......... .......... .......... .......... .......... ..........
HERV-K108       .......... .......... .......... .......... .......... .......... .......... .......... .......... ..........
HERV-K109       .......... .......... .......... .......... .....----- -----..... .......... .......... .......... ..........
HERV-K115       .......... .......... .......... .......... .......... .......... .......... .......... .......... ..........
AP00076         .......... .....L.... .......... .......... .......... .......... .......... .......... .H........ ..........
AC025420        .......... .......... .......... .......... .......... .......... .......... ...L...... .......... ..........
Y17833          ..V....... .......... .......... .......... .......... .......S.. .....I.... .......... .......... ..........
Consensus       .......... .......... .......... .......... .......... .......... .......... .......... .......... ..........
oriHERV-K113    .......... .......... .......... .......... .......... .......... .......... .......... .......... ..........

                      710        720        730        740        750        760        770        780        790        800
                ....|....| ....|....| ....|....| ....|....| ....|....| ....|....| ....|....| ....|....| ....|....| ....|....|
HERV-K113       MGVPEKIKTD NGPGYCSKAF QKFLSQWKIS HTTGIPYNSQ GQAIVERTNR TLKTQLVKQK EGGDSKECTT PQMQLNLAPY TLNFLNIYRN QTTTSAEQHL
HERV-K101       .......... .......... .......... .......... .......... .......... .......... ........L. .......... ..........
HERV-K102       .......... .......... .......... R......... .......... .......... .......... ........L. .......... ..........
HERV-K104       .......... .......... .......... .......... .......... ..N.--.... .......... ........L. .......... ..........
HERV_K107       .......... .......... .......... .......... .......... .......... .......... ........L. .......... ..........
HERV-K108       .......... .......... .......... .......... .......... .......... .......... ........L. .......... ..........
HERV-K109       .......... .......... .......... .......... .......... .......... .......... ........L. .......... ..........
HERV-K115       .......... .......... .......... .......... .......... .......... .......... ........L. .......... ..........
AP00076         .......... .......... .......... .......... .......... .......... .......... ........L. .......... ..........
AC025420        .......... .......... .......... .......... .......... .......... .......... ........L. ........G. ..........
Y17833          .......... .......... .......... .......... .......... .......... .......... ........L. .......... ..........
Consensus       .......... .......... .......... .......... .......... .......... .......... ........L. .......... ..........
oriHERV-K113    .......... .......... .......... .......... .......... .......... .......... ........L. .......... ..........

                      810        820        830        840        850        860        870        880        890        900
                ....|....| ....|....| ....|....| ....|....| ....|....| ....|....| ....|....| ....|....| ....|....| ....|....|
HERV-K113       TGKKNSPHEG KLIWWKDNKN KTWEIGKVIT WGRGFACVSP GENQLPVWMP TRHLKFYNEP IGDAKKSTSA ETETPQSSTV DSQDEQNGDV RRTDEVAIHQ
HERV-K101       .......... .......... .......... .......... ........I. .......... ......RA.T .--------- ---------- ----------
HERV-K102       .......... .......... .......... .......... ........I. .......... ......RA.T .--------- ---------- ----------
HERV-K104       ..*....... .......... .......... .......... ........I. .......... .......... ....L..... .......... ..R.......
HERV_K107       .......... .......... .......... *......... ........L. .......... ......RA.T .--------- ---------- ----------
HERV-K108       .......... .......... .......... .......... ........I. .......... .R........ ....S..... .......... ..........
HERV-K109       .......... .......... .......... .......... ........I. ..P....... .R........ .......... .......... ..........
HERV-K115       .......... .......... .......... .......... ........I. .......... .R........ .......... .......... ..........
AP00076         .......... .......... .......... .......... ........I. .......... .R........ .......... .......... ..........
AC025420        .......... .......... .......... .......... ........I. ........A. .......... .......... .........I X.........
Y17833          .......... .......... .......... .......... ........I. .......... .......... .......... .......... ..........
Consensus       .......... .......... .......... .......... ........I. .......... .R........ .......... .......... ..........
oriHERV-K113    .......... .......... .......... .......... ........I. .......... .......... .......... .......... ..........
```

```
                        910        920        930        940        950
                 ....|....| ....|....| ....|....| ....|....| ....|....| ....|..
HERV-K113        EGRAADLGTT KEADAVSYKI SREHKGDTNP REYAACSLDD CINGGKSPYA CRSSCS*
HERV-K101        ---------- ---------- ---------- ---------- ---------- ------
HERV-K102        ---------- ---------- ---------- ---------- ---------- ------
HERV-K104        .......... .......... .......... .......... .......... ......*
HERV_K107        ---------- ---------- ---------- ---------- ---------- ------
HERV-K108        .....N.... .......... .......... .......... .......... ......*
HERV-K109        .......... .......... .......... .......... .......... ......*
HERV-K115        .......... .......... .......... .......... .......... ......*
AP00076          .....N.... .......... .......... .......... .......... ......*
AC025420         .......... .......... .......... .......... .......... ......*
Y17833           .S........ .......... .......... ......G... .......... ......*
Consensus        .......... .......... .......... .......... .......... ......*
oriHERV-K113     .......... .......... .......... .......... .......... ......*
```

- Deletion (Nucleotide
Insertion(Nucleotide)
* Stopcodon
▒ Mutation
□ Variation
⋁ Insertion (Amino Acid)

Additional File 1B

DNA Sequence of oricoHERV_K113 GagProPol

```
       KpnI
      -----+
   1  ggtaccgaat tcgtcgacga gagatcccga gtacgtctac agtcagcctt acggtaagct

  61  tgtgcgctcg gaagaagcta gggtgataat gggccagacc aagagcaaga tcaagagcaa

 121  gtacgccagc tacctgagct tcatcaagat cctgctgaag aggggcggag tgaaggtgtc

 181  caccaagaac ctgatcaagc tgttccagat catcgagcag ttctgcccct ggttccccga

 241  gcagggcacc ctggacctga aggactggaa gcggatcggc aaagagctga agcaggccgg

 301  caggaagggc aacatcatcc ccctgaccgt gtggaacgac tgggccatca tcaaggccgc

 361  cctggaaccc ttccagaccg aagaggacag cgtgagcgtg agcgacgccc ctggcagctg

 421  catcatcgac tgcaacgaga aaacgcgtaa gaagagccag aaagagaccg agagcctgca

 481  ctgcgagtac gtggccgagc ccgtgatggc ccagagcacc cagaacgtgg actacaacca

 541  gctgcaggaa gtgatctacc ccgagaccct gaagctggaa ggcaagggcc ccgaactggt

 601  gggccccagc gagagcaagc ccaggggcac cagccctctg cctgccggcc aggtgcccgt

 661  gaccctgcag ccccagaaac aggtgaaaga gaacaagacc cagcccccccg tggcctacca

 721  gtactggccc cctgccgagc tgcagtaccg gcctcccccc gagagccagt acggctaccc

 781  cggcatgccc cctgcccctc agggcagagc cccctacccc cagcctccca ccaggcggct

 841  gaaccccacc gcccctccca gcaggcaggg cagcgagctg cacgagatca tcgacaagag

 901  caggaaagag ggcgacaccg aggcctggca gttccctgtg accctggaac ccatgcctcc

 961  cggcgagggc gcccaggaag gcgagccccc caccgtggag gcccggtaca agagcttcag

1021  catcaagatg ctgaaggaca tgaaagaagg cgtgaagcag tacggcccca acagccccta

1081  catgcggacc ctgctggaca gcatcgccca cggccaccgg ctgatccctt acgactggga

1141  gatcctggcc aagagcagcc tgagccccag ccagttcctg cagttcaaga cctggtggat

1201  cgacggcgtg caggaacagg tgcggcggaa cagggccgcc aacccccccg tgaacatcga

1261  cgccgaccag ctgctgggca tcggccagaa ctggtccacc atcagccagc aggccctgat

1321  gcagaacgag gccatcgagc aggtgcgggc catctgcctg cgggcctggg agaagatcca

1381  ggaccccggc agcacctgcc ccagcttcaa caccgtgaga cagggcagca aagagcccta

1441  ccccgacttc gtggcccggc tgcaggacgt ggcccagaag agcatcgccg acgagaaggc

1501  ccggaaggtg atcgtggagc tgatggccta cgagaacgcc aaccccgagt gccagagcgc

1561  catcaagccc ctgaagggca aggtgccagc cggcagcgac gtgatcagcg agtatgtgaa
```

1621 ggcctgcgac ggcatcggcg gagccatgca caaggccatg ctgatggccc aggccatcac

1681 cggcgtggtg ctgggcggac aggtgcggac cttcggcggc aagtgctaca actgcggcca

1741 gatcggccac ctgaagaaaa actgccccgt gctgaacaag cagaacatca ccatccaggc

1801 caccaccacc ggcagagagc cccccgacct gtgcccccgg tgcaagaagg gcaagcactg

1861 ggccagccag tgcagaagca agttcgataa aaatgggcaa ccattgtcgg gaaacgagca

1921 aaggggccag cctcaggccc cacaacaaac tggggcattc ccaattcagc catttgttcc

1981 tcagggtttt cagggacaac aacccccact gtcccaagtg tttcagggaa taagccagtt

2041 accacaatac aacaattgtc ccccgccaca agcggcagtg cagcagtaga tctgtgcaca

2101 atccaggccg tgagcctgct gcccggcgag cctccccaga agatccccac cggcgtgtac

2161 ggccctctgc ccgagggcac cgtgggcctg atcctgggcc ggtccagcct gaacctgaag

2221 ggcgtgcaga tccacactag tgtggtggac agcgactaca agggcgagat ccagctggtg

2281 atcagcagca gcatcccctg gtccgccagc cctggcgacc ggatcgccca gctgctgctg

2341 ctgccctaca tcaagggcgg caacagcgag atcaagcgga tcggcggcct gggcagcacc

2401 gaccccacag gcaaggccgc ctactgggcc tcccaggtgt ccgagaaccg gcccgtgtgc

2461 aaggccatca tccagggcaa gcagttcgag ggcctggtgg acacaggcgc cgacgtgagc

2521 atcatcgccc tgaaccagtg gcccaagaac tggcccaagc agaaagccgt gaccggcctg

2581 gtgggcatcg ggaccgccag cgaggtgtac cagagcacag agatcctgca ctgtctgggc

2641 cccgacaacc aggaaagcac cgtgcagccc atgatcacca gcatccccct gaatctgtgg

2701 ggcagggacc tgctgcagca gtggggagcc gagatcacca tgcctgcccc cctgtacagc

2761 cccacaagcc agaaaatcat gaccaagatg ggctacatcc ccggcaaggg cctgggcaag

2821 aacgaggacg gcatcaaagt gcccgtggag gctaaaataa atcaaaaaag agaaggaata

2881 gggtatcctt tttaggggcg gccactgtag agcctcctaa acccatacca ttaacttgga

2941 aaacagaaaa accagtgtgg gtaaatcagt ggccgctacc taagcagaaa ctggaagccc

3001 tgcacctgct ggccaacgaa cagctggaaa agggccacat cgagcccagc ttcagcccct

3061 ggaacagccc cgtgttcgtg atccagaaga aaagcggcaa gtggcggatg ctgaccgacc

3121 tgcgggccgt gaacgccgtg atccagccca tgggcccccct gcagcctggc ctgcccagcc

3181 ccgccatgat ccccaaggac tggcctctga tcatcatcga tctgaaggac tgcttcttca

3241 ccatccccct ggccgagcag gactgcgaga agttcgcctt taccatcccc gccatcaaca

3301 acaaagagcc cgccacccgg ttccagtgga aggtgctgcc ccagggcatg ctgaacagcc

3361 ccaccatctg ccagaccttc gtgggcagag ctctgcagcc agtgagagag aagtttagcg

3421 actgctacat catccactac atcgacgaca tcctgtgtgc cgccgagacc aaggacaagc

3481 tgatcgattg ctacaccttc ctgcaggccg aggtggccaa tgccggcctg gccatcgcca

3541 gcgacaagat ccagaccagc accccccttcc actacctggg catgcagatc gagaaccgga

3601 agatcaagcc tcagaagatc gagatcagga aggacaccct gaaaaccctg aacgacttcc

3661 agaagctgct gggggacatc aactggatcc ggcccaccct gggcatcccc acctacgcca

3721 tgagcaacct gttcagcatc ctgcggggcg acagcgacct gaacagcaag agaatgctga

3781 cccccgaggc caccaaagaa atcaaactgg tggaggaaaa gatccagagc gcccagatca

3841 acaggatcga ccccctggcc cctctgcagc tgctgatctt cgccaccgcc cacagcccta

3901 ccggcatcat catccagaac accgatctgg tggagtggag cttcctgccc cacagcaccg

3961 tgaaaacctt caccctgtac ctggaccaga tcgccaccct gatcggccag acccggctgc

4021 ggatcatcaa gctgtgcggc aacgaccccg acaagatcgt ggtgcccctg accaaagaac

4081 aggtgcgcca ggccttcatc aacagcggcg cctggcagat cgggctggcc aatttcgtgg

4141 gcatcatcga taaccactac cccaagacca agatcttcca gttcctgaag ctgaccacct

4201 ggatcctgcc caagatcaca cggcgggagc ctctggaaaa cgccctgacc gtgttcaccg

4261 acggcagcag caacggcaaa gccgcctata ccggccccaa agaacgggtg atcaagaccc

4321 cctaccagtc cgcccagcgg gccgaactgg tggccgtgat caccgtgctg caggacttcg

4381 accagcccat caacatcatc agcgacagcg cctacgtggt gcaggccacc cgggacgtgg

4441 agaccgccct gatcaagtac agcatggacg accagctgaa ccagctgttc aatctgctgc

4501 agcagaccgt gcggaagcgg aacttcccct tctacatcac ccacatccgg gcccacacca

4561 acctgccagg ccctctgacc aaggccaatg agcaggccga cctgctggtg tccagcgccc

4621 tgattaaggc ccaggaactg cacgccctga cacacgtgaa tgccgccgga ctgaagaaca

4681 agttcgacgt gacctggaag caggccaagg acatcgtgca gcactgcacc cagtgccagg

4741 tgctgcacct gcccacccag gaagccggcg tgaaccccag gggcctgtgc cccaacgccc

4801 tgtggcagat ggacgtgacc cacgtgccca gcttcggcag actgagctac gtgcacgtga

4861 ccgtggacac ctacagccac ttcatctggg ccacctgcca gaccggcgag agcaccagcc

4921 acgtgaagaa acacctgctg tcctgcttcg ccgtgatggg cgtgcccgag aagatcaaga

4981 ccgacaacgg ccctggctac tgcagcaagg ccttccagaa gttcctgagc cagtggaaga

5041 tctcccacac cacaggaatt ccttacaaca gccagggcca ggccatcgtg gagcggacca

5101 accggacact gaaaacacag ctggtgaagc agaaagaggg gggcgactcc aaagagtgca

5161 ccaccccccca gatgcagctg aacctggccc tgtacaccct gaactttctg aacatctacc

5221 ggaaccagac caccacctcc gccgagcagc acctgaccgg caagaagaac agcccccacg

5281 agggcaagct gatttggtgg aaggacaaca agaataagac ctgggagatc ggcaaggtga

5341 tcacctgggg gagaggtttt gcttgtgttt caccaggaga aaatcagctt cctgtttgga

```
5401  tacccactag acatttgaag ttctacaatg aacccatcgg agatgcaaag aaaagcacct

5461  ccgcggagac ggagacaccg caatcgagca ccgttgactc acaagatgaa caaaatggtg

5521  acgtcagaag aacagatgaa gttgccatcc accaagaagg cagagccgcc gacttgggca

5581  caactaaaga agctgacgca gttagctaca aaatatctag agaacacaaa ggtgacacaa

5641  accccagaga gtatgctgct tgcagccttg atgattgtat caatggtggt aagtctccct

                                           XhoI
                                         -+-----
5701  atgcctgcag gagcagctgc agctaactcg ag
```
